# Popular media as a double-edged sword: An entertainment narrative analysis of the controversial Netflix series *13 Reasons Why*

**DOI:** 10.1371/journal.pone.0255610

**Published:** 2021-08-11

**Authors:** Hua Wang, Juliet J. Parris

**Affiliations:** Department of Communication, University at Buffalo, The State University of New York, Buffalo, NY, United States of America; Charles Sturt University - Port Macquarie Campus, AUSTRALIA

## Abstract

*13 Reasons Why* is a Netflix original series adapted from Jay Asher’s 2007 young adult novel with the same title. Season 1 premiered on March 31, 2017 and featured the sensitive issue of teen suicide along with bullying, substance use, depression, and sexual assault. Unlike the typical teen dramas on popular streaming platforms, this show was created not only for entertainment, but also to stimulate conversations about taboo topics that people often shy away from. However, it also caused significant controversy, especially criticism around the main character Hannah’s suicide scene. More than three years into the initial controversy and at least two dozen scholarly publications later, this study is the first to examine the entertainment narrative content of *13 Reasons Why* Season 1 to better understand how these health and social issues were portrayed in the show, what specific examples we could identify as potential behavioral modeling, and to what degree it complied with the 2017 WHO guidelines for media professionals. We used the framing theory and social cognitive theory in communication research and media studies as our guiding conceptual frameworks and a narrative analysis approach to investigate a total of 660 cut scenes in all 13 episodes. Our findings provided empirical evidence, along with contextual information and detailed examples, to demonstrate that a popular entertainment program like the Netflix series *13 Reasons Why* serves as a double-edged sword. The production team’s good will and due diligence are commendable. Yet, additional steps can be taken in the future to effectively promote professional resources and reduce viewers’ risks, especially the most vulnerable groups.

## Introduction

Suicide is a serious global health concern, accounting for an annual loss of nearly 800,000 lives [1]. In the United States, approximately 48,000 people take their lives each year and millions more report suicidal ideation, plans, and attempts [2, 3]. Although the United States does not have the biggest share of deaths from suicide in the world [4], suicide has been climbing at an alarming rate in recent decades [5] with an increase of 33% nationwide from 1999 to 2019 [2]. Suicide also affects Americans of all ages, especially the younger groups [6, 7]. From 2007 to 2018, suicide rates for the 10-24-year-olds increased by a staggering 57% and across 42 out of 50 states [8]. During this period, suicide had replaced homicide as the second leading cause of death for the 20–24 age group since 2010, for the 15–19 age group since 2011, and for the 10–14 age group since 2014 [6].

The causes of suicide are complex. Years of research have shown that childhood trauma, genetic vulnerability, psychological distress, mental health disorders, bullying, substance use, sexual violence, and firearm access are among the most salient suicide risk factors [9–16]. Suicide-related thoughts and behaviors are emotionally draining for both the individual themselves and their loved ones [17]. The associated financial costs to the society could easily amount to billions of dollars [18–21]. For example, Mrazek and colleagues calculated the burden of major depression alone to be $106-$118 billion from 1996 to 2013 [19]. Shepard and colleagues estimated the total cost of suicides and suicide attempts at $93.5 billion, an equivalent of $298 per capita, in 2013 [20]. Based on a 6:1 benefit-cost ratio, they recommended increasing investment in prevention efforts such as medical and counseling services [20]. Similarly, Feldman and colleagues suggested that public health initiatives need to be cost-effective yet uncompromising of high quality to improve mental health and prevent suicides [13].

Media reporting of suicide can be difficult to balance between raising awareness among the public while protecting the well-being of those at risk, with common concerns of suicide contagion or the copycat effect [9, 22, 23]. This is getting even more complex and challenging with highspeed internet and social media consumption among adolescents and young adults [24, 25]. Poor media portrayal of suicide is not only irresponsible but also harmful to youths [9, 22, 24, 25]. In addition to formal and informal channels of news media, entertainment programs in popular culture can also exert tremendous social influence on sensitive issues like suicide. It is an understudied area yet can offer invaluable insights for media praxis and scholarship. Therefore, the purpose of this study was to examine the controversial series *13 Reasons Why* using a narrative analysis approach.

*13 Reasons Why* is an original teen drama series on the popular streaming platform Netflix. Season 1 was adapted from Jay Asher’s 2007 young adult novel with the same title and premiered on March 31, 2017. The story was narrated from the perspective of the main character, Hannah Baker, a 17-year-old American high schooler who died from suicide and left behind 13 cassette tapes explaining the 13 reasons why she killed herself. The narrative developments were also driven by another main character, Clay Jensen, who cared deeply about Hannah and wanted to find out what happened to her. Unlike the typical teen dramas, *13 Reasons Why* was created to not only entertain the audience with its dramatic twists and turns in a fictional world but also stimulate conversations in real life about the taboo topic of teen suicide that people often shy away from. The producers went so far as to include a 29-minute companion documentary entitled, *13 Reasons Why*: *Beyond the Reasons*, featuring the cast, producers, and expert consultants. They discussed how difficult decisions were made during production and promoted an affiliated website (www.13ReasonsWhy.info) with resources on suicide prevention and mental health. The program was quickly dubbed “the most tweeted-about show” after the season premiere [26, 27] and renewed by Netflix for a second season [28].

However, it also caused significant controversy, especially criticism around Hannah’s suicide scene [29–31]. Mainstream media coverage on *13 Reasons Why* warned that suicide prevention experts advise against describing suicide in such graphic detail due to the concern of suicide contagion [32]. Some news reports headlined stories such as “Families blame *13 Reason Why* for teen daughters’ suicides” [33], pointing to the Netflix series as a trigger for the two teenage girls who watched the show and died four days apart. One of the teens’ uncle stated that the two-minute suicide scene of Hannah Baker was too graphic. This scene was eventually removed [34]. More than three years into the initial controversy and at least two dozen scholarly publications later, other than blatant blames and conceptual arguments [35–37], not a single study has analyzed exactly how teen suicide was portrayed in *13 Reasons Why*.

As part of a larger research project, we conducted an in-depth investigation of *13 Reasons Why* Season 1 using a narrative analysis approach. Our study conceptualization was guided by Entman’s framing theory in mass media research [38] and Bandura’s social cognitive theory used in the scholarship and praxis of entertainment-education [39, 40]. We examined all 13 episodes in Season 1, with cut scenes as our units of analysis and a theme-based inquiry focusing on major health and social issues portrayed in the narrative. In addition, we identified examples of positive and negative behavioral modeling and checked the media portrayal in *13 Reasons Why* against the WHO’s guidelines for media coverage of suicide [41].

In this article, we begin with a brief literature review on the two aforementioned theoretical frameworks along with the rationale that led to our research questions. We then provide descriptions of the entertainment content and our narrative analysis before detailing the results. We conclude with a discussion about the major findings, implications, and recommendations for future endeavors in research and practice.

## Literature review

### Message framing in mass media

The notion of framing was initially developed by Goffman as a way to help people organize and process information in everyday life [42]. Gitlin further connected framing with mass media in the context of news coverage when reporters employ frames to draw public attention to certain aspects of a phenomenon [43]. Over the last four decades, framing has been regarded as a major theory of mass communication and media effects [44, 45]. Entman explicated that “to frame is to select some aspects of a perceived reality and make them more salient in a communicating text, in such a way as to promote a particular problem definition, causal interpretation, moral evaluation, and/or treatment recommendation for the item described” (p. 52) [38]. Therefore, the two essential elements of framing are *selection* and *salience*, which are the focus of the present study. It is important because what aspects of a phenomenon and how they are presented in the mass media can have significant psychological, political, and even societal impacts [46–50]. Message framing helps to reduce the complexity of issues, making them more accessible to the audiences by highlighting certain aspects of the content and playing to their existing schemas [51]. In mass media, television news frame events from a particular viewpoint, which may change the viewers’ perceptions. The way health and social issues are framed in entertainment media and popular culture can influence the audience, especially the younger groups who may be vulnerable and underprepared for exposure to sensitive content without proper warning or guidance. For example, DuRant and colleagues found that tobacco and alcohol use was frequently glamorized in music videos and portrayed as socially desirable by the lead artists [52]. Collins and colleagues showed in a national longitudinal survey that exposure to sexual content in entertainment programming on television was a predictor of adolescent sexual initiation [53].

Many other media initiatives purposefully leverage the power of storytelling and address important issues through entertainment media by framing their message in unconventional ways [54–56]. For example, the protagonist in a popular Indian television crime series *Jasoos Vijay* (Detective Vijay) revealed that he was HIV positive and used this dramatic twist to help reframe the public discourse, raise awareness, and reduce sigma around HIV/AIDS [56]. Brusse and colleagues demonstrated when using a gain (as opposed to loss) frame in health messages about alcohol consumption, the Dutch participants reported a decrease in counterarguing and a higher level of intention for refraining from drunk cycling [54]. In the United States, institutions such as the Norman Lear Center has been monitoring health messaging in primetime television for decades; they provide expert consultation to creative writers and recognize outstanding work through the Sentinel Health Award every year [57, 58]. Based on the framing theory and empirical studies reviewed above, we proposed these research questions:

**RQ1:** What health and social issues were portrayed in the Netflix series *13 Reasons Why*?
**RQ2:** How were these health and social issues portrayed in Netflix series *13 Reasons Why*?

### Entertainment-education as enabling media

For decades now, entertainment media for mass audiences have been used to raise awareness, increase knowledge, spur discussions, shift attitudes, and influence practice using a social and behavior change communication strategy called entertainment-education [56, 58–60]. Bandura’s social cognitive theory (formerly known as social learning theory) [39] has been the most prominent theory for entertainment-education scholars and practitioners [61, 62]. Most people know about the famous bobo doll experiment that helped Bandura develop the social learning theory in the 1960s and 1970s in the context of understanding the media effects of violent behavioral modeling on children. In particular, the bobo doll experiment demonstrated the power of role modeling and observational learning when the modeled behavior was filmed rather than in real life (Singhal as quoted in Friedman [63]).

In fact, since the 1970s, this theory has also heavily influenced the design and research of numerous entertainment-education interventions around the world [61, 62]. A wide range of novel, effective, and healthy behaviors have been portrayed by popular characters in the entertainment-education radio and television dramas to facilitate positive individual and collective behavior change [56, 58, 60]. One exemplary project is *Soul City* in South Africa. In Season 4 of their popular television drama serial, *Soul City* modeled a collective action as the bystander intervention to curb domestic violence (i.e., neighbors banging pots and pans to demonstrate their disapproval of the abusive husband beating up his wife) and enabled villagers around the country to follow suit [58]. In the United States, primetime television producers have worked with expert consultants to tackle the controversy of abortion such as in Norman Lear’s *Maude* [59] or promote organ donation through behavior modeling in the medical drama, *Numb3rs* [64]. Bandura called such entertainment-education dramatic serials “enabling media” [40], which help create conditions for activating human agency and facilitating positive change.

In that sense, with their producers’ explicit intention to spur public discussions about taboo topics and their documented efforts to consult with subject experts (as shown in the 29-minute companion documentary), the Netflix series *13 Reasons Why* may be viewed as an incidental entertainment-education drama series to address teen suicide and other related issues. However, good intentions do not necessarily guarantee good outcomes. The concerns surrounding *13 Reasons Why* are certainly legitimate regarding its potential unintended negative influence on vulnerable and high-risk viewers. To avoid repeating the same mistakes, we ought to acknowledge the efforts made by the production team while taking a deep dive to investigate incidents that may be perceived as positive vs. negative behavioral modeling. Well-respected international and national health organizations have laid out guidelines for media coverage of suicide. For example, the WHO provides a resource with a list of six do’s and six don’ts for media professionals when reporting on suicide [41]. It is imperative to comply with these established guidelines. Therefore, we proposed two additional research questions:

**RQ3:** Which incidents in *13 Reasons Why* may be considered as positive vs. negative behavioral modeling for its audience?
**RQ4:** Did *13 Reasons Why* comply with the WHO’s guidelines for covering the issue of suicide in mass media?

## Materials and methods

### Sample description

A total of 660 cut scenes across all 13 episodes of *13 Reasons Why* Season 1 were analyzed in this study, ranging from 40 to 68 scenes per episode (*M* = 50.77, *SD* = 8.21). Each cut scene, by definition, had a distinctive portrayal of an event that happens at one location at one point in time. If the time or location changed, it was counted as a separate scene. Each episode lasted about 50–60 minutes and featured a person who was, as Hannah explained in the cassette tape, a reason why she killed herself (Table 1). The entire season accumulated a total of 43,512 seconds (or 12.09 hours). The length of each episode varied from 2,999 to 3,710 seconds (*M* = 3347.08, *SD* = 214.88). There were 19 teen characters, 14 parent characters, and six educator characters, often with multiple characters appearing in a single cut scene (Table 2). The stories took place in many of the teen characters’ private residences, various locations at school, and popular spots in the local community (Table 3). Out of 642 scenes with a clear indication about the time of the day, 388 (58.8%) were daytime scenes and 254 (38.5%) were nighttime scenes.

**Table 1:** Netflix series *13 Reasons Why* Season 1 summary by episodes.

Episode #	Featured Character	Duration (in seconds)	Number of Scenes
1	Justin Foley	00:54:03 (3,243)	45
2	Jessica Davis	00:51:52 (3,112)	52
3	Alex Standall	00:57:23 (3,443)	48
4	Tyler Down	00:57:20 (3,440)	43
5	Courtney Crimsen	00:58:56 (3,536)	40
6	Marcus Cole	00:52:09 (3,129)	41
7	Zach Dempsey	00:53:51 (3,231)	68
8	Ryan Shaver	00:54:07 (3,247)	54
9	Justin Foley	00:58:56 (3,536)	59
10	Sheri Holland	00:49:59 (2,999)	46
11	Clay Jensen	00:54:35 (3,275)	54
12	Bryce Walker	01:01:50 (3,710)	60
13	Mr. Porter	01:00:11 (3,611)	50

*Note*. The episode durations and cut scenes are based on the original version when *13 Reasons Why* Season 1 was released in 2017. They do not account for any subsequent modifications (such as the addition of Public Service Announcements before episodes start or the removal of sensitive scenes) that might have changed the data.

**Table 2:** Netflix series *13 Reasons Why* Season 1 cut scenes by characters.

No.	Teen Characters		Parent Characters		Educator Characters	
1.	Clay Jensen	365	Mrs. Baker	52	Mr. Porter	40
2.	Hannah Baker	208	Mr. Baker	26	Mrs. Bradley	16
3.	Justin Foley	107	Mrs. Jensen	41	Principal Bolan	9
4.	Jessica Davis	105	Mr. Jensen	21	Coach Patrick	5
5.	Zach Dempsey	83	Mrs. Dempsey	5	Vice Principal Childs	2
6.	Tony Padilla	75	Mr. Crimsen (Todd)	4	Mrs. Antilly	1
7.	Alex Standall	67	Courtney’s other dad	1		
8.	Bryce Walker	57	Mr. Davis	3		
9.	Courtney Crimsen	54	Mrs. Davis	1		
10.	Sheri Holland	45	Mrs. Down	2		
11.	Marcus Cole	42	Mr. Down	2		
12.	Tyler Down	32	Ms. Foley	2		
13.	Ryan Shaver	18	Ms. Foley’s boyfriend	1		
14.	Montgomery	15	Mr. Padilla	1		
15.	Jeff	12				
16.	Stephanie	6				
17.	Ashley	5				
18.	Kat	4				
19.	Pratter	3				
	**Total:**	**1,303**	**Total:**	**162**	**Total:**	**73**

*Note*. The total number of cut scenes in Season 1 was 660. Each cut scene may have more than one character.

**Table 3:** Netflix series *13 Reasons Why* Season 1 cut scenes by locations.

Scenes at Private Locations		Scenes at School		Scenes at Other Public Locations	
Clay Jensen’s house	66	School: Hallway	48	Street/outdoors	54
Hannah Baker’s house	53	School: Gym	32	Monet’s coffee shop	30
Jessica Davis’ house	45	School: Classroom	31	The Bakers’ Drugstore	17
Bryce Walder’s house	20	School: Mr. Porter’s office	29	Crestmont Theatre	14
Justin Foley’s house	11	School: Outside/parking lot	21	Rosie’s Diner	9
Tony’s car	7	School: Cafeteria	14	Deposition meeting	8
Alex Standall’s house	6	School: Main office	11	Blue Spot Liquor Store	7
Tyler Down’s house	6	School: Winter formal	8	Eisenhower Park	6
Jeff Atkin’s house	5	School: Courtyard	7	Hannah’s gravesite	4
Courtney Crimsen’s house	3	School: Locker room	6	Poetry group	4
Zach Dempsey’s house	3	School: Library	4	Police station	3
Tony Padilla’s house	3	School: Auditorium	2	Mrs. Jensen’s office	2
Hannah’s jeep	2	School: Hannah’s locker	2	Sugaring Hair Salon	2
Sheri’s car	2	School: Girls’ bathroom	2	Walplex pharmacy	2
The Cantrells’ house	2	School: Boy’s bathroom	1	Restaurant	1
Jessica’s car	1	School: Dark room	1		
The Porters’ house	1	School: Music room	1		
		School: Principal’s office	1		
		School: Vice Principal’s office	1		
**Total:**	**236**	**Total:**	**223**	**Total:**	**163**

*Note*. The total number of cut scenes in Season 1 with valid location information was 622.

### Narrative analysis

We adopted a narrative analysis approach in this study to better understand the entertainment content in *13 Reasons Why* Season 1. Narrative analysis is an analytic approach in social and behavioral sciences with a focus on the interpretation of information conveyed in the form of stories, and it has been used in social psychology, communication research, and media studies [65, 66]. Scholars have examined a wide range of elements from character development and overarching themes to narrative structure and dramatic performance, but these choices are usually made according to the study aims and research inquiries [65].

Television dramas are a popular narrative genre in entertainment media with tremendous values for research because they are an indispensable part of the popular culture and can have a significant impact on individual audiences, social norms, and even public policy [56, 67–70]. For example, Freytag and Ramasubramanian used narrative analysis of 113 deaths portrayed in four popular medical dramas to examine the attributes of characters who died, their causes of death, the underlying themes in these death storylines, and their (dis)connections with the “good death” experiences in reality [71].

Similar to the approach used by Freytag and Ramasubramanian [71], all episodes in our narrative analysis of *13 Reasons Why* Season 1 were coded through multiple iterations. The initial rounds of coding involved identifying cut scenes in each episode, marking their starting and ending time, detailing the characters who appeared, their specific locations, time of the day when the event took place, and connections to any health and social issues. Knowing that the producers of *13 Reasons Why* were well-intentioned but many news reporters and scholars disagreed on its audience response in reality, we reviewed the storylines and character dialogues closely based on each major health and social issue portrayed in the show to identify illustrative behavioral modeling examples for potential positive and negative impact.

In addition, we compared the entertainment narrative content against the WHO guidelines for media coverage of suicide [41]. Based on a comprehensive literature review, field experiences, and expert recommendations, they provided a list of six dos and six don’ts regarding the type of information that can be helpful and the language and details to avoid. We created exclusionary criteria based on how the issue of teen suicide was portrayed in the narrative. From the do list, we selected three of six items where the issue was present, for example “Do provide accurate information about where to seek help”. We excluded the other three items since they were not relevant to how teen suicide was portrayed such as “Do apply caution when reporting celebrity suicides”. Similarly, from the don’t list, we selected three of six items where the issue of teen suicide was present, but was not responsibly reported, such as “Don’t use language which sensationalizes or normalizes suicide, or presents it as a constructive solution to problems”. We excluded the other three items since they were not relevant to the narrative, such as “Don’t use photographs, video footage or social media links”. After agreeing on the list of do and don’t’ items, we compared them against the 65 related scenes covering teen suicide. All relevant scenes were reviewed multiple times and coded in three categories: WHO guidelines complied, not complied, and not applicable.

## Results

### What were portrayed and how

RQ1 and RQ2 asked what specific health and social issues were portrayed in *13 Reasons Why* Season 1 and how. As shown in Table 4, the series covered several important issues in a total of 141 cut scenes: teen suicide (65 scenes, 46.1%), bullying (29 scenes, 20.6%), substance use (29 scenes, 20.6%), depression (12 scenes, 8.5%), and sexual assault (6 scenes, 4.3%). They were spread out across the entire Season 1, accounting for 21.4% of the entire air time. Every single one of the 13 episodes portrayed between two to five of these issues in multiple scenes (*Range* = 5–20, *M* = 10.85, *SD* = 5.01). This does validate the producers’ intention about raising social awareness through storytelling to stimulate difficult dialogues in the American society.

**Table 4:** Netflix series *13 Reasons Why* Season 1 cut scenes by issues.

Episodes	Teen Suicide	Bullying	Substance Use	Depression	Sexual Assault	Total
1	4	3	0	2	0	9
2	3	5	0	3	0	11
3	9	7	1	1	1	19
4	7	5	4	0	0	16
5	4	3	2	2	0	11
6	0	2	1	0	0	3
7	17	0	1	2	0	20
8	6	0	1	1	0	8
9	3	0	7	0	2	12
10	2	1	5	0	0	8
11	2	0	3	0	0	5
12	1	1	3	0	3	8
13	7	2	1	1	0	11
**Total**	**65**	**29**	**29**	**12**	**6**	**141**

Our in-depth analysis also showed that each of these five themes was addressed from different angles. Teen suicide included five sub-themes: awareness of the connection between depression and suicide (6 scenes, 9.2%), the early signs of Hannah’s suicidal ideation (2 scenes, 3.1%), Hannah’s premeditation (3 scenes, 4.6%), Hannah’s death by suicide (6 scenes, 9.2%), and coping with Hannah’s death (48 scenes, 73.8%). Bullying included five sub-themes: spreading rumors and gossips at school (7 scenes, 24.1%), invasion of privacy through stalking (3 scenes, 10.3%), physical abuse (6 scenes, 20.7%), verbal abuse (8 scenes, 27.6%), and cyberbullying (5 scenes, 17.2%). Substance use included two sub-themes: alcohol consumption such as underage and binge drinking (20 scenes, 69.0%) and marijuana use (9 scenes, 31.0%). Depression included three sub-themes: symptom recognition (9 scenes, 75.0%), teaching about symptoms (1 scene, 8.3%), and general knowledge (2 scenes, 16.7%). Sexual assault included two sub-themes: sexual harassment (5 scenes, 83.3%) and rape (1 scene, 16.7%).

As shown in Table 5, issue-related narrative elements centered around three types of characters: 18 teen characters in 360 scenes, eight parent characters in 62 scenes, and five educator characters in 21 scenes. Not surprisingly, among the most frequently appeared characters were the people closely connected to Hannah’s suicide, including the people she named in the cassette tapes she left behind but also Clay’s friend Tony. In particular, Clay was in 24.4% of the scenes, and Hannah was in 13.1% of the scenes related to the health and social issues. This was consistent with the way the series was presented, with Clay driving the suspense and Hannah being the narrator of her story. The Netflix series also added more weight to the parent characters as compared to the original novel, especially around Hannah’s and Clay’s parents. Such screenplay adaptation brought another critical layer of parent-children communication in addition to the peer-to-peer interactions among the teens. A few educator characters were also included. The counselor Mr. Porter was in 13 scenes as he was featured in Hannah’s last tape and also the presenter of the coping workshop that school organized for parents after Hannah’s death. Other characters such as Mrs. Bradley were featured in classroom scenes sharing suicide prevention resources with the students. Through them, the dramatic narrative incorporated the perspectives and dialogues between the educators and the teen students.

**Table 5:** Netflix series *13 Reasons Why* Season 1 cut scenes by characters (issue-related scenes only).

No.	Teen Characters		Parent Characters		Educator Characters	
1.	Clay Jensen	88	Mrs. Baker	22	Mr. Porter	13
2.	Hannah Baker	47	Mrs. Jensen	14	Mrs. Bradley	3
3.	Justin Foley	37	Mr. Baker	11	Principal Bolan	3
4.	Jessica Davis	32	Mr. Jensen	10	Coach Patrick	1
5.	Alex Standall	27	Ms. Foley’s boyfriend	2	Mrs. Antilly	1
6.	Zach Dempsey	25	Mrs. Dempsey	1	Vice Principal Childs	0
7.	Bryce Walker	25	Ms. Foley	1	**Total:**	**21**
8.	Tony Padilla	14	Mr. Davis	1		
9.	Courtney Crimsen	14	Mrs. Davis	0		
10.	Tyler Down	11	Mrs. Down	0		
11.	Marcus Cole	10	Mr. Down	0		
12.	Montgomery	10	Mr. Crimsen (Todd)	0		
13.	Sheri Holland	9	Courtney’s other dad	0		
14.	Jeff	4	Mr. Padilla	0		
15.	Ryan Shaver	3	**Total:**	**62**		
16.	Pratter	2				
17.	Stephanie	1				
18.	Ashley	1				
19.	Kat	0				
	**Total:**	**360**				

*Note*. We kept the complete list of character names from the sample description to reduce confusion. Therefore, any character who appeared in Season 1 but not in the issue related cut scenes will show 0 count in this table.

As shown in Table 6, issue-related events portrayed in this show were divided between 66 scenes (46.8%) in private such as homes and cars, and 92 scenes (53.2%) in public such as hallways at school and the Monet’s coffee shop in the community. Moreover, out of 141 issue-related cut scenes with a clear indication about the time of the day, 86 (61.0%) were daytime scenes and 55 (39.0%) were nighttime scenes.

**Table 6:** Netflix series *13 Reasons Why* Season 1 cut scenes by locations (issue-related scenes only).

Private Locations		School Locations		Other Public Locations	
Hannah Baker’s house	21	School: Hallway	15	Monet’s coffee shot	6
Clay Jensen’s house	18	School: Classroom	9	The Bakers’ Drugstore	7
Bryce Walder’s house	11	School: Gym	7	Street/outdoors	4
Jessica Davis’ house	10	School: Mr. Porter’s office	7	Blue Spot Liquor Store	3
Justin Foley’s house	4	School: Outside/parking lot	6	Deposition meeting	2
Alex Standall’s house	1	School: Main office	5	Police station	1
Tony’s car	1	School: Winter formal	4	Hannah’s gravesite	1
Tyler Down’s house	0	School: Courtyard	4	Eisenhower Park	1
Jeff Atkin’s house	0	School: Locker room	3	Crestmont Theatre	1
Courtney Crimsen’s house	0	School: Cafeteria	1	Walplex pharmacy	0
Zach Dempsey’s house	0	School: Girl’s bathroom	1	Sugaring Hair Salon	0
Tony Padilla’s house	0	School: Hannah’s locker	1	Rosie’s Diner	0
The Cantrells’ house	0	School: Vice Principal’s office	1	Restaurant	0
The Porters’ house	0	School: Music room	1	Poetry group	0
Hannah’s jeep	0	School: Different locations	1	Mrs. Jensen’s office	0
Sheri’s car	0	School: Library	0		
Jessica’s car	0	School: Auditorium	0		
		School: Boy’s bathroom	0		
		School: Principal’s office	0		
		School: Dark room	0		
**Total:**	**66**	**Total:**	**66**	**Total:**	**26**

*Note*. We kept the complete list of location names from the sample description to reduce confusion. Therefore, any location that appeared in Season 1 but not in the issue related cut scenes will show 0 count in this table.

Taken together, Table 7 summarizes all issue-related scenes across the five themes, three types of characters, three types of locations, and two time periods of the day. Although these numbers are mere proxies of different ways the producers attempted to address these complex and difficult issues, these results provide more nuanced and contextual information on how the dramatic narrative was structured to tell the stories and prompt the audiences to engage in deeper reflections and open discussions.

**Table 7:** Netflix series *13 Reasons Why* Season 1 percentage of characters, locations, time of day (issue-related scenes only).

Issues	Characters			Location			Time of Day	
	*Teens*	*Parents*	*Educators*	*School*	*Private*	*Other*	*Daytime*	*Nighttime*
**Teen Suicide**	54	19	11	30	17	18	42	23
	(64.3%)	(23.6%)	(13.1%)	(46.2%)	(26.2%)	(27.7%)	(64.6%)	(35.4%)
**Bullying**	25	5	6	17	4	8	22	7
	(71.1%)	(13.2%)	(15.8%)	(58.6%)	(13.8%)	(27.6%)	(75.9%)	(24.1%)
**Substance Use**	29	2	1	10	16	3	11	18
	(90.6%)	(6.3%)	(3.1%)	(34.5%)	(55.2%)	(10.3%)	(37.9%)	(62.1%)
**Depression**	12	2	4	9	3	0	12	0
	(66.7%)	(11.1%)	(22.2%)	(75.0%)	(25.0%)	(0.0%)	(100.0%)	(0.0%)
**Sexual Assault**	6	0	0	0	4	2	1	5
	(100.0%)	(0.0%)	(0.0%)	(0.0%)	(66.6%)	(33.3%)	(16.7%)	(83.3%)

### Potential issue-related behavioral modeling

RQ3 asked which incidents in *13 Reasons Why* may be considered as positive vs. negative behavioral modeling for its audience. Based on our review of the 141 issue-related cut scenes, we identified one positive example and one negative example for each of the five themes to illustrate their behavioral modeling potential (Table 8). Episode 4 Scene 8 was a good example to tackle teen suicide. In a few short lines, Clay’s dad acknowledged that he understood his son was grieving a dear friend in pain, showing a parent’s sincere concern and encouraging Clay to express his feelings. This kind of parent-child communication is critical for a teenager to cope with death from suicide. Similar approaches of being present and showing support as a parent can also help reduce a child’s risk for psychological distress, mental health disorders, and ultimately suicide. On the other hand, Episode 13 Scene 6 was an example of negative behavior modeling. Showing any details about suicidal plans in terms method and location can increase the risk for the audience, especially those who are already highly susceptible.

**Table 8:** Examples of potential positive and negative behavioral modeling in *13 Reason Why* Season 1.

Issue	Positive	Negative
**Teen Suicide**	Episode 4, Scene 8	Episode 13, Scene 6
	Time Stamp: 9:47–11:39	Time Stamp: 4:17–5:28
	Clay wakes up in bed with a hangover from the night before. As he struggles to cope with Hannah’s death, his father, Mr. Jensen said to him, "You gotta start opening up to us, kid. Just you know, let us know what’s going on. . .”	Hannah goes into her parents’ drugstore. She didn’t want to engage in a conversation, just used the excuse to get one more cassette tape for her school project to pick up a box of razor blades and walked out.
**Bullying**	Episode 10, Scene 27	Episode 1, Scene 40
	Time Stamp: 27:07–28:29	Time Stamp: 43:18–44:20
	While Tyler tries to convince Sheri that they should go talk to Clay, Montgomery approaches them and aggressively slams Tyler into the lockers. Right in that moment, Alex intervenes and demands Montgomery to back off.	In the hallway, Justin brags to friends about his hot date Hannah by showing them an intimate picture of her in the park at night on his phone. Bryce takes away the phone and shares it with the entire school, leading others to spread rumors about Hannah.
**Substance Use**	Episode 9, Scene 9	Episode 10, Scene 41
	Time Stamp: 3:20–4:37	Time Stamp 41:19–41:28
	Zach, Sheri, Justin, Bryce, and Jessica are chatting before class. Jessica reaches for a flask that contains alcohol, and her boyfriend, Justin, questions her decision by asking, “Since when do you drink at school?”	Clay recounts the events from the night of the party that his friend Jeff died. Although Jeff didn’t drink, beer bottles were found in his car. In fact, it was Sheri who drove under the influence and knocked down a stop sign that caused the accident.
**Depression**	Episode 4, Scene 9	Episode 1, Scene 3
	Time Stamp: 11:40–12:33	Time Stamp: 3:18–4:04
	Mr. Porter and Principal Bolton are hosting a meeting with concerned parents to educate them about symptoms of depression, such as mood swings, refusal to participate in group activities, change in appearance, and substance abuse.	Clay has a flashback to when Hannah cut her hair short, which he didn’t recognize then but now knows that a sudden change in her appearance was a sign of her struggle with depression.
**Sexual Assault**	Episode 11, Scene 44	Episode 12, Scene 35
	Time Stamp: 36:46–39:09	Time Stamp: 41:39–43:27
	While at Jessica’s party, Hannah and Clay go into Jessica’s bedroom to talk. After a while, they start making out, and as things heat up between the two, Clay asks, “This, okay?” in which Hannah consents by saying, “Yes, more than okay!”	Hannah walks into Bryce’s party where underage drinking was occurring. She sees Jessica, her ex-best friend, who invites her into the hot tub. Hannah agrees by stripping down into her underwear and joins the party. Jessica and Justin leave, and Bryce joins Hannah alone in the hot tub. Unwarranted, Bryce sexually assaults Hannah as she struggles out of the tub.

*Note*. The cut scene number and time stamps are all based on the original version of Season 1 released in 2017 and do not reflect any subsequent content modifications that might have affected the starting and ending time of selected cut scenes.

Episode 10 Scene 27 was a good example of bullying bystander intervention that Alex decisively stepped in to stop Montgomery’s physical aggression towards Tyler. Unfortunately, bullying is common in high school and Montgomery’s character represented student athletes who are seen as strong and popular sometimes become bullies. Episode 1 Scene 40 was an example of negative behavior, showing how easy it could be for an innocent joke to spin out of control and turn into cyberbullying.

Episode 9 Scene 9 was a good example related to substance use, showing when Jessica tried to numb her pain by hiding alcohol in her drinks at school during the day, her boyfriend Justin gently nudged her to reconsider her decision. Episode 10 Scene 41 recounted how, in a series of events from a night of partying, that drunk driving can cause the life of a good friend.

Episode 4 Scene 9 was a good example related to depression, showing a school assembly where the school counselor and principal discuss symptoms of depression, suicide and coping strategies with concerned parents. It included specific and scientific medical information. Episode 1 Scene 3 showed a flashback when Hannah was clearly suffering from depression but no one around her recognized. It may be considered as a counter example of the previous one, as the lack of awareness about Hannah’s suffering eventually failed her.

Episode 11 Scene 44 was a good example illustrating that young people can be sexually responsible while having a good time when Clay obtained Hannah’s consent before moving further in their intimate moment. Episode 12 Scene 35, on the other hand, showed how an easy-going party could become so wrong when Bryce sexually assaulted Hannah in the hot tub, exacerbating her pain and suffering.

For any of these selected cut scenes from *13 Reasons Why* Season 1, there is the potential of reducing negative influence and facilitating positive change, if the modeled behaviors were unique, novel, and effective as a convincing alternative to the existing norm, a warning message was displayed before or after the episode, and they were all directly linked to proper resources as these narrative elements emerged in the folding storylines.

### Compliance with WHO guidelines

RQ4 asked if *13 Reasons Why* Season 1 complied with the WHO guidelines for portraying suicide in the media. Table 9 summarizes the results based on our review of the 65 suicide-related scenes. First, responsible professional practices do provide accurate information about where to seek help. The recommendation aims to disseminate resources about social services and support network, including suicide prevention centers, crisis helplines, other health and welfare professionals in public discussions about suicide. Access to support is also vital when providing information. In our study, 9.2% of the suicide-related scenes did report accurate information about where students could go to seek help. For example, Episode 1 had several scenes portraying Mrs. Bradley telling the students in class where they could find information on suicide prevention if they or someone they cared about needed help.

**Table 9:** Selected scenes from Netflix series *13 Reasons Why* for WHO guideline compliance.

DOs	DON’Ts
(1) DO provide accurate information about where to seek help.	(1) DON’T use language which sensitizes or normalizes suicide, or presents it as a constructive solution to problems.
Episode 1, Scene 2	Episode 7, Scene 4
Time Stamp: 2:51–3:17	Time Stamp: 1:35–2:30
Mrs. Brandley informs her students about where to locate information on getting help if needed for themselves or a friend. A student challenges her in which Mrs. Bradley reinforces the importance of discussing the topic to properly recognize “the signs that someone you care for needs help.”	Mr. & Mrs. Jensen have conflicting views about Clay returning to Talk Therapy. Mrs. Jensen worries that with the constant emails, themed “Contagion, Suicide Clusters” from the school and Clay not communicating with them that he might be in trouble.
(2) DO educate the public about the facts of suicide and suicide prevention, without spreading myths.	(2) DON’T explicitly describe the method used for suicide.
Episode 4, Scene 9	Episode 13, Scene 6
Time Stamp: 11:40–13:25	Time Stamp: 4:21–4:59
Mr. Porter coordinated an informative presentation with Principal Bolton to educate concerned patients about suicide prevention and symptoms to recognize within their children such as moodiness, change of appearance, declining GPA.	While grabbing another cassette from her parent’s store, Hannah also secretly takes a pack of razor blades before telling her mother she is off.
(3) DO report stories of how to cope with life stressors or suicidal thoughts, and how to get help.	(3) DON’T provide details about the site or location of suicide.
Episode 7, Scene 3	Episode 13, Scene 35
Time Stamp: 1:43–2:41	Time Stamp: 35:50–37:10
Mrs. Jensen worries about her son’s behavior, so she tries to urge her husband that they “need to get him back to Dr. Ellman” so that Clay has support if needed.	In the present, Clay is in Mr. Porter’s office describing Hannah’s suicide. Concurrently, "[Hannah] got into the tub still with her clothes on slit her wrists and bled to death. And she died alone."

Second, responsible professional practices do educate the public about the facts of suicide and suicide prevention without spreading myths. This recommendation suggests that since there are many misconceptions about suicide, it is important to lead reports with facts than myths to avoid imitative behaviors. It is also advisable to report on suicide prevention and 75.4% of the suicide-related scenes in our study did exactly that. For example, Episode 4 had several scenes about the suicide prevention workshop the high school offered to concerned parents.

Third, responsible professional practices to share stories of how to cope with life stressors or suicidal thoughts. This recommendation involves providing personal narratives that integrate educational materials describing various coping strategies to build psychological resilience and restore hope. These stories usually feature unique ways in which others overcome adversity. In our study, 10.7% of the suicide-related scenes did portray these narrative elements. For example, Episode 7 included scenes where Clay’s parents demonstrated positive role modeling. His father shared his own high school experiences with him and how he survived the difficult time while his mother is willing to get him the necessary help with a physician.

Moreover, we also identified the incidents where the suicide-related scenes in *13 Reasons Why* violated the WHO guidelines. In fact, 12.0% of these scenes included language that could potentially normalize suicide or present it as a solution to problems. Also, out-of-context use of the word “suicide” is destructive and should be avoided as it may desensitize the weight of the issue when reported to the public. A common example is the expression “committing suicide”, which criminalizes the behavior. The topic of “suicide contagion” and “suicide clusters” were included in the conversation between Clay’s parents but the connotation of infectious disease may be expressions of concern by some and should be avoided [9].

In addition, 9.0% of the suicide-related scenes described the method used. The WHO urges media professionals to be cautious when it comes to the method of suicide because it may trigger copycat behavior, especially some stories can spread like a wild fire on social media. In our study, the guideline was violated when details of Hannah’s suicide plans played out on the screen, including picking up razor blades from her parents’ drugstore in Episode 13.

Worse yet, 6.0% of the suicide-related scenes provided details about the site or location. Responsible media professionals would avoid including information about the site or location of the suicide to reduce the risk for vulnerable populations. Instead, care is recommended to avoid promoting or glamorizing such sites or locations. The most criticized scene of Season 1 was the elaborate scene of Hannah taking her own life in the bathtub at home in Episode 13 and was eventually removed from the original version due to a strong backlash from suicide prevention advocates over its graphic nature.

## Discussion

In this study, we examined the entertainment content of the controversial Netflix original series *13 Reasons Why* Season 1 using a narrative analysis approach. We aimed to better understand how teen suicide and other related health and social issues were portrayed in the show, what specific examples we could identify as potential behavioral modeling, and to what degree it complied with the WHO guidelines for media professionals [41]. Our results suggest that in a 12-hour entertainment program, on average, more than one in every five scenes in *13 Reasons Why* Season 1 (a total of 141 out of 660 scenes, 21.4%) included narrative elements to tackle a critical issue. The five health and social issues featured themes were: teen suicide (46.1%), bullying (20.6%), substance use (20.6%), depression (8.5%), and sexual assault (4.3%). Each of the 13 episodes covered at least two if not all five of these themes. Each issue was portrayed through a whole host of key characters, storylines, and sub-themes.

We learned that issue-related scenes about teen suicide (46.2%), bullying (58.6%), and depression (75.0%) occurred at school; substance abuse (55.2%) and sexual assault (66.6%) occurred mainly in private homes. In addition, 61.0% of the issue-related scenes occurred during the day, while substance abuse (62.1%) and sexual assault (83.3%) occurred more often at night. These results are consistent with previous research. For example, there is a close linkage between suicide and adolescents suffering from major depressive disorder, bullying, and sexual assault [11]. With increasingly pervasive technologies and the popularity of social media, cyberbullying has detrimental effects to suicide amongst teens [11, 16]. Most sexual assault victims were attacked in the evening and while at home [72].

Overall, the issue-related scenes were organized around three types of characters: driven by 18 teen characters and supported by eight parent characters and five educator characters; the events mostly took place between private residences at night or school facilities and other popular public locations in the community during the day. A great majority of these issue-related portrayals were honest, intentional, and educational. Most criticism in the mainstream news reports and scholarly publications have only focused on the one scene of Hannah’s suicide in the bathtub and not any of the other 140 scenes. And to date, there has not been any acknowledgement or appreciation for the production team’s effort to consult with the subject experts while making difficult creative decisions, as documented in the show’s companion program *Beyond the Reasons*.

Moreover, parents (44.0%) and educators (14.9%) were included in many of the issue-related scenes as part of this Netflix original series, although they didn’t carry much weight in the 2007 novel. These efforts were well-intended and captured in our research findings, although the response in reality from many adult viewers of the show were not necessarily positive, especially regarding how the school counselor, Mr. Porter, was portrayed as the last reason why Hannah couldn’t find any alternative but to end her own life. Criticism of Mr. Porter’s character was linked to the lack of mental health resources established by Hannah’s school as she sought help before her suicide [35, 73]. This muting effect or dismissive behavior highlighted the need for better trained professionals and counseling services in public schools where students spend most of their days [35, 73].

Our narrative analysis also revealed that there were multiple examples for each of the five major issues that could potentially serve as behavioral modeling. Teen characters, parent characters, and educator characters were all included in the positive examples, such as peer support, parent-child communication, and school-based prevention efforts. Most of them could be easily strengthened based on entertainment-education principles to facilitate positive change, while a few others could also be seen as reinforcing current teen cultural norms that might lead to health and social problems.

When checking the 65 suicide-related scenes against the WHO guidelines [41], we found a cumulative of 95.3% of these scenes were in compliance with what WHO recommended to DO, with the best efforts in educating the public about suicide prevention (75.4%) followed by recognizing suicidal thoughts and developing coping strategies (10.7%) and providing accurate information about resources for seeking help (9.2%). We also found a cumulative of 27.7% of these suicide-related scenes to have violated the DON’Ts in the WHO guidelines [41], with the worst part of using language that might sensitize or normalize suicide (12.3%) followed by detailing the method (9.2%) or the site/location (6.2%).

These findings are qualitatively insightful in response to our research questions. However, we want to acknowledge that the theme-based coding procedures were not completed without any challenge during the iterative process of our narrative analysis. Disagreements occurred mainly when certain characters appeared in a particular scene without speaking a word. We decided to make an exception for inclusion if a character showed nonverbal expressions that were a meaningful part of the narrative in that scene. For example, Bryce winked at Hannah in the hallway at school as if nothing happened between them although he actually sexually assaulted Hannah just the other night at his private party. In rare incidents like this, even though Bryce did not speak in this scene, his nonverbal expression was relevant to the issue of sexual assault and ultimately contributed to Hannah’s suicide. Therefore, Bryce was included in our calculation of characters in this scene. In other scenes such as a teacher discussing school resources for suicide prevention while Hannah’s grieving parents walk by the classroom door on their way to their daughter’s locker, since the narrative focus of this scene was about the discussion in the classroom, the non-speaking characters were not included in the final coding. Once any discrepancies in the initial rounds of coding were resolved and the focal themes of teen suicide, bullying, substance use, depression, and sexual assault were decided, scenes connected to the focal themes were watched and coded again to examine the types of characters featured, contextual factors like location and time, as well as specific angles or aspects used to portray each theme. Our analytical procedures and final codes are available through OSF (https://bit.ly/3xLKFaZ).

For the first time, our findings provide empirical evidence, along with contextual information and detailed examples, to support the arguments made by scholars such as Mueller [36] and Krebs [35] that a popular entertainment program like the Netflix series *13 Reasons Why* serves as a double-edged sword. This study is also timely, with the COVID-19 pandemic magnifying many risk factors (e.g., economic distress, social isolation, lack of mental health resources), many have deep concerns about suicide rates and the physical, emotional, financial, and societal toll that will take on our communities around the globe [74–76]. Although these trends were relatively stable in the early months of pandemic lockdowns, [6, 74, 77, 78], some have reported that COVID-19 increase risks linked to suicide among the youth groups [79].

## Conclusion

Our study filled in a critical gap in the scholarly investigations of the controversial Netflix series *13 Reasons Why* by providing a detailed account of its media portrayal of teen suicide and related issues of bullying, substance use, depression, and sexual assault. The production team’s goodwill and due diligence are commendable. Unfortunately, good intentions and efforts cannot guarantee positive audience response as one would hope. Additional steps can be taken to enhance effective behavioral modeling in the media portrayal, offer warning messages before and/or after viewing, and promote professional resources directly and promptly linked to the specific health and social issues during and/or immediately after viewing.

Results of this study will be compared with how the show was covered in mainstream news media to explore the similarities and differences on the focus of framing in the entertainment narratives as opposed to the content of news reports about this series. Taken together, the lessons learned from the controversy stirred by *13 Reasons Why* will help both the entertainment producers and the public, especially young audiences, their parents, educators, and other adult viewers, to better understand how to discuss difficult health and social issues sensibly, responsibly, and effectively.
